# Nationwide Implementation of Non-Mandatory Preventive Medicine Programmes in Japanese Municipalities: A Descriptive Cross-Sectional Survey and Evidence–Practice Gap Analysis

**DOI:** 10.21203/rs.3.rs-9674474/v1

**Published:** 2026-05-12

**Authors:** Hideki Mori, Kazuhiro Shimomura, Kei Miyazaki, Kazuya Honda, Ayako Shibata, Masatsugu Sakata, Masaki Futamura, Yusuke Tsugawa, Kenta Murotani, Kei Mukohara

**Affiliations:** NHO Nagasaki Medical Center; Aichi Cancer Center Hospital; Nagoya City University Graduate School of Medical Sciences; Junshin Gakuen University (Nurse Practitioner Course); Yodogawa Christian Hospital; Nagoya City University Graduate School of Medical Science; NHO Nagoya Medical Center; UCLA Fielding School of Public Health; Kurume University; Kurume University

**Keywords:** preventive medicine, municipal health policy, evidence–practice gap, Japan, cross-sectional survey, non-mandatory screening, evidence-based prevention, Health policy, Implementation research, Municipal health services, USPSTF, Screening, Cross-sectional study, Overdiagnosis

## Abstract

**Background.:**

In Japan, much preventive medicine outside mandated national programmes is left to municipal discretion, yet the nationwide alignment between these locally administered programmes and graded evidence remains unexamined. We mapped implementation of non-mandatory preventive medicine programmes across Japanese municipalities and quantified evidence–practice gaps.

**Methods.:**

A nationwide cross-sectional survey was administered to all 1,741 Japanese municipalities between March and August 2025. Implementation (excluding the five mandated cancer screenings under the Health Promotion Act) was defined as municipal provision or subsidisation and calculated on municipality-count and population-weighted bases. Each programme was benchmarked against USPSTF recommendation grades, and an Evidence–Practice Alignment (EPA) score was derived for each municipality (weights: A = + 2, B = + 1, D = − 2, I or ungraded = − 1). Non-response bias was assessed by comparing responding and non-responding municipalities using standardised mean differences derived from national statistical databases.

**Results.:**

Valid responses were received from 467 municipalities (response rate, 26.8%) across all 47 prefectures. Implementation rates ranged from 0.2% to 92.1% (municipality-count) and 0.0% to 94.4% (population-weighted). Ten Grade A/B programmes had implementation below 50%, including folic acid supplementation (3.2%), syphilis screening (9.6%), and abdominal aortic aneurysm screening (16.1%). Conversely, hepatitis B/C (92.1%, 91.4%) and osteoporosis screening (68.5%) were widely implemented. Several Grade D or ungraded programmes showed appreciable uptake, notably young adult health checkups (86.3%; ungraded), brain/carotid screening (41.1%; Grade D), and frailty screening (26.1%; ungraded). Population-weighted coverage exceeded municipality-count rates for HIV (48.1% vs 10.1%) and syphilis (48.3% vs 9.6%) screening, indicating concentration in larger municipalities. Prefectural EPA scores ranged from − 4 to 1, with heterogeneity and no clear geographic gradient.

**Conclusions.:**

Substantial evidence–practice gaps and equity concerns coexist in Japan’s municipal preventive medicine programmes. Our findings support strengthened dissemination of graded evidence to municipal decision-makers and critical reassessment of low-value screening.

## Background

Ageing populations and rising healthcare expenditures have placed preventive healthcare at the centre of health policy agendas worldwide [1, 2], yet the translation of evidence-based recommendations into consistent population-level practice remains an enduring challenge across healthcare systems. Japan represents a particularly illuminating case. Preventive healthcare in Japan is organised through a layered structure involving national legislation, medical insurers, and municipal governments, with the Ministry of Health, Labour and Welfare (MHLW) playing a central role in setting policy frameworks and guidelines [3]. Some programmes are mandated by national law: the Specific Health Check-up and Specific Health Guidance (Tokutei Kenshin), for example, are required under the Act on Assurance of Medical Care for Elderly People and are delivered by medical insurers to individuals aged 40–74 enrolled in public health insurance schemes [4]. Notably, even some components of these nationally mandated programmes do not always align with contemporary international evidence standards, suggesting that questions of evidence–practice alignment extend across the full spectrum of preventive services in Japan, both mandated and non-mandated. Other services are delegated to municipalities under the Health Promotion Act, the Maternal and Child Health Act, and related legislation. For these municipally delivered services—which particularly serve residents not covered by employer-based health check-ups—national policies provide general guidance, but the actual implementation, including the types of screenings offered, target populations, and outreach strategies, varies substantially across municipalities. Such locally administered programmes fall broadly into two types: those for which national guidelines exist but implementation authority rests with municipalities (as in cancer screening), and those developed entirely at the local level without national policy guidance (as in carotid artery stenosis or COPD screening). While cancer screening programmes are relatively well-monitored through national surveillance data on uptake [5, 6], a systematic cross-sectional evaluation of the full spectrum of municipally administered preventive medicine programmes—beyond the five nationally designated cancers—and their alignment with current scientific evidence remains lacking.

One widely referenced benchmark is the US Preventive Services Task Force (USPSTF) in the United States, which provides one of the world’s most structured and methodologically rigorous evidence-grading frameworks for clinical preventive services [7]. However, even within the United States, substantial evidence gaps, implementation barriers, and health inequities persist, particularly among underserved populations, indicating that a centralized evidence-grading framework alone does not guarantee equitable or optimal preventive care delivery [8]. In contrast, Japanese municipalities lack a comparable cross-cutting evidence-grading framework for non-mandatory preventive services, which may contribute to heterogeneous implementation [3]. Against this background, this study had two objectives: first, to describe how non-mandatory preventive medicine programmes are implemented by municipalities across Japan; and second, to evaluate how well these programmes align with a structured evidence-based benchmark, and to identify gaps between evidence and practice.

## Methods

### Study design

A descriptive cross-sectional study was conducted using a questionnaire survey administered to all 1,741 municipalities in Japan. Implementation of non-mandatory preventive medicine programmes was described based on data from responding municipalities. We excluded the five cancer screenings mandated under the Health Promotion Act (gastric, colorectal, lung, breast, and cervical), but included non-mandatory cancer-related screenings such as thyroid ultrasound and positron emission tomography (PET) cancer screening that are delivered or subsidised at municipal discretion. Reporting followed STROBE and CHERRIES guidelines [16, 17].

### Study population

The study population comprised all municipalities in Japan (n = 1,741), defined as cities, towns, villages, and the 23 special wards of Tokyo. Administrative wards within designated cities were excluded, as they function as internal subdivisions rather than independent local government units. Questionnaires were distributed to the health check-up and screening divisions (or equivalent responsible department) of all municipalities as a census survey. The analytical sample consisted of those returning completed responses. Municipalities were included if they submitted responses via web-based form, Microsoft Word document, or PDF, and were excluded if identification was not possible or if more than 50% of items were missing or duplicated.

### Data collection

Data collection was conducted from 10 March to 31 August 2025. Questionnaires were distributed by postal mail to the health check-up and screening divisions of all municipalities, and participation was voluntary. Respondents could complete the survey via a password-protected web-based form (Google Forms), or by returning a completed PDF or Word document by mail. No financial compensation was provided; a summary of findings was offered to all responding municipalities upon study completion. Responses were managed at the municipal level using password and municipality identifier systems to prevent duplicate submissions; cookie- or IP-based verification was not performed. The majority of web-based items were designated as required fields. Where PDF responses contained missing data, municipalities were contacted and data supplemented where feasible.

### Survey items

The survey covered the implementation status of non-mandatory preventive medicine programmes, excluding vaccinations and the five mandated cancer screenings. Target items were selected based on programmes with documented implementation records in Japan and USPSTF recommendations. The questionnaire comprised 60 items across 11 pages and used primarily mandatory multiple-choice formats to ensure completeness and reduce respondent burden, supplemented by open-ended fields.

### Definition of implementation

Implementation was defined as municipal provision or subsidisation of a given screening test or service, irrespective of whether the target age, sex, or risk criteria specified in USPSTF recommendations were met. Detailed eligibility criteria could not be collected for most items—exceptions included osteoporosis screening and brain dock—and implementation was therefore recorded on a binary basis. This approach was adopted to maintain response rates and enable consistent classification across municipalities.

### External data

Municipal baseline characteristics were obtained from public governmental databases—including e-Stat (System of Social and Demographic Statistics), the Geospatial Information Authority of Japan, and Ministry of Health, Labour and Welfare sources—as well as official municipal websites. Derived indicators included total population, area, population density, ageing rate, Rurality Index for Japan, age-adjusted mortality rate, fiscal capacity index, income per capita, proportion of residents exempt from resident tax, and numbers of hospitals, clinics, and physicians [9-13].

### Data management

All response data were centrally managed by the research team. Web-based responses were processed via automatically generated spreadsheets; postal responses were manually entered according to a standardised protocol. Open-ended responses were standardised against predefined definitions and coded as categorical variables where applicable. Data cleaning was performed according to pre-established criteria to ensure analytical reproducibility.

### Variable definitions

Each preventive medicine programme was coded as a binary variable (1 = implemented or subsidised; 0 = otherwise). USPSTF grades (A, B, D, or I) were assigned to each item based on recommendations current as of March 2025, the time of survey administration [7]. We selected the USPSTF framework as a structured external benchmark because, although Japan has multiple domain-specific preventive health policies and guidelines, it lacks a single cross-cutting, nationally authoritative evidence-grading system for many non-mandatory preventive services. The USPSTF was chosen because it provides one of the most methodologically rigorous and internationally recognized frameworks for evaluating preventive services across diverse domains using explicit assessments of net benefit and harm. Our use of USPSTF grades was not intended to imply direct transferability of US recommendations to the Japanese context, but rather to provide a transparent and standardized reference framework for comparative evaluation of municipal policy alignment across heterogeneous preventive programmes. Items not evaluated by the USPSTF were classified as ungraded. For Grade A or B items, a municipality was classified as implementing the programme if it provided or subsidised the service in any form.

### Evidence–Practice Alignment (EPA) score

The EPA score was calculated as a weighted sum of implementation status across all items. Weights were assigned a priori by the study team to reflect the relative strength of evidence conveyed by each USPSTF grade: A = + 2, B = + 1, D = − 2, and I or ungraded = − 1; non-implementation of a programme was assigned 0 points. No prior validated scoring instrument exists for this purpose; the weighting scheme was therefore developed de novo to penalise implementation of services with evidence of net harm (Grade D) while treating insufficient-evidence items (Grade I) and ungraded items conservatively. Higher scores indicate greater implementation of recommended services alongside appropriate restraint regarding non-recommended ones; lower scores reflect the inverse.

### Descriptive analysis and evidence–practice gap

Implementation rates for each programme were calculated descriptively by municipality count and by population-weighted proportion, then compared against USPSTF grades to identify programmes with low uptake among Grade A/B items and high uptake among Grade D, I, or ungraded items, thereby characterising evidence–practice gaps.

### Non-response bias assessment

To evaluate self-selection bias, baseline characteristics of responding and non-responding municipalities were compared using external data obtained from public governmental databases. Although baseline characteristics are presented in Table 2 as medians and interquartile ranges to reflect the skewed distributions of municipal-level variables, standardised mean differences (SMDs) were calculated using the mean and standard deviation of each variable, in accordance with the conventional formula. Between-group differences were quantified using SMDs, with ∣SMD∣ < 0.10 indicating adequate balance, 0.10–0.20 minor imbalance, and > 0.20 meaningful imbalance [14, 15]. Given the descriptive intent of the study and the impossibility of fully characterising the non-response mechanism from observed covariates, we did not apply statistical adjustments for non-response. Instead, the assessment of potential bias was limited to comparisons of observed characteristics between responding and non-responding municipalities.

### Missing data

Missing data were minimised by designating the majority of web-based items as required fields. Residual missing values in postal responses were addressed through direct follow-up with municipalities.

### Use of Large Language Models

During the preparation of this manuscript, the authors used ChatGPT (OpenAI, GPT-4) and Claude (Anthropic) to assist with translation from Japanese to English and to improve the clarity and readability of draft text. All AI-assisted outputs were critically reviewed, edited, and verified by the authors, who take full responsibility for the content of the manuscript. No AI tool was used to generate original scientific content, perform data analysis, draw conclusions, or generate references.

## Results

### Respondent municipality profile

#### Response rate and geographic distribution

Responses were received from 468 of 1,741 municipalities; one municipality with a wholly missing questionnaire was excluded, yielding 467 valid responses (response rate, 26.8%). Responding municipalities were distributed across all 47 prefectures, encompassing urban, peri-urban, and rural areas. In terms of population size, five municipalities (1.1%) had populations of 1,000,000 or more, 78 (16.7%) had 100,000–999,999, 248 (53.1%) had 10,000–99,999, and 136 (29.1%) had fewer than 10,000 residents; municipalities with fewer than 1,000,000 residents accounted for 462 (98.9%) of respondents (Table 1).

Prefecture-level response rates were calculated with the number of responding municipalities as the numerator and the total number of municipalities within each prefecture as the denominator; 95% confidence intervals were estimated using the Wilson score method (Additional file 1: Table S1). Response rates by region (8-region classification) are shown as % (n/N) in Additional file 2: Table S2. Prefecture-level response rates ranged from 9.3% to 48.8%, with a median of 26.7% (interquartile range [IQR] 20.8–31.9%). The geographic distribution of response rates was visualised using a choropleth map (Additional file 3: Figure S1). A funnel plot with the overall response rate (26.8%) as the reference line and 95% and 99.8% control limits, shown in Additional file 4: Figure S2, demonstrated that between-prefecture variation in response rates persisted even after accounting for statistical fluctuation associated with denominator size.

#### Characteristics of responding and non-responding municipalities

Baseline characteristics of responding (n = 467) and non-responding municipalities were compared using external data, and standardised mean differences (SMDs) were calculated (Table 2). The Rurality Index for Japan (RIJ) and ageing rate were well balanced between groups (RIJ: 56 [IQR 34–79] vs 54 [30–77]; ageing rate: 36.4% [30.1–40.9] vs 35.5% [29.8–41.7]), suggesting no substantial imbalance. Only two variables exceeded the prespecified threshold for adequate balance: per-capita resident income (3,088,363 JPY [2,894,575–3,354,483] vs 3,121,105 JPY [2,883,839–3,399,850]; SMD = 0.124) and general clinic physician density (809.64 [332.73–2,154.56] vs 1,147.11 [462.28–3,004.44]; SMD = 0.104), indicating that responding municipalities tended to be slightly lower-income and have fewer clinic physicians than non-responding municipalities. All other variables, including the fiscal capacity index, total population, and hospital density, were adequately balanced (all ∣SMD∣ < 0.10).

### Main analysis

#### Implementation rates of non-mandatory preventive medicine programmes

Implementation rates of non-mandatory preventive medicine programmes were calculated on both a municipality-count basis (programme penetration) and a population-weighted basis (population coverage) (Table 3).

#### Municipality-count-based (unweighted) implementation rates

Unweighted implementation rates varied substantially across programmes, ranging from 0.2% to 92.1%. HBV and HCV screening were implemented by more than 90%, and young adult health check-ups by 86.3%, whereas unhealthy drug use screening, IPV screening, and gonorrhoea screening were rarely implemented. Osteoporosis screening was also relatively widespread (68.5%), while depression/anxiety screening remained uncommon (3.2%).

#### Population-weighted implementation rates

Population-weighted implementation rates ranged from 0.0% to 94.4%. Programmes with the highest population coverage were HCV screening (94.4%), HBV screening (93.6%), young adult health check-ups (78.8%), and osteoporosis screening (70.2%). Programmes with the lowest population coverage were unhealthy drug use screening (0.0%), cardiac screening by echocardiography (0.3%), IPV screening (0.8%), PAD screening by ankle–brachial index (0.9%), and perinatal depression screening (1.2%).

#### Divergence between municipality-count and population-weighted rates

For certain programmes, population-weighted coverage was substantially higher than municipality-count-based rates. HIV screening (10.1% by municipality count vs 48.1% population-weighted), syphilis screening (9.6% vs 48.3%), and chlamydia screening (7.7% vs 36.6%) were implemented by relatively few municipalities, yet population-weighted coverage was comparatively high, suggesting that these services tend to be provided in larger municipalities. Conversely, for brain/carotid screening (41.1% vs 35.6%) and young adult health check-ups (86.3% vs 78.8%), municipality-count-based penetration exceeded population-weighted coverage, suggesting that these programmes are implemented disproportionately in smaller municipalities.

#### Evidence–practice gaps against USPSTF recommendation grades

**Figure 1:**
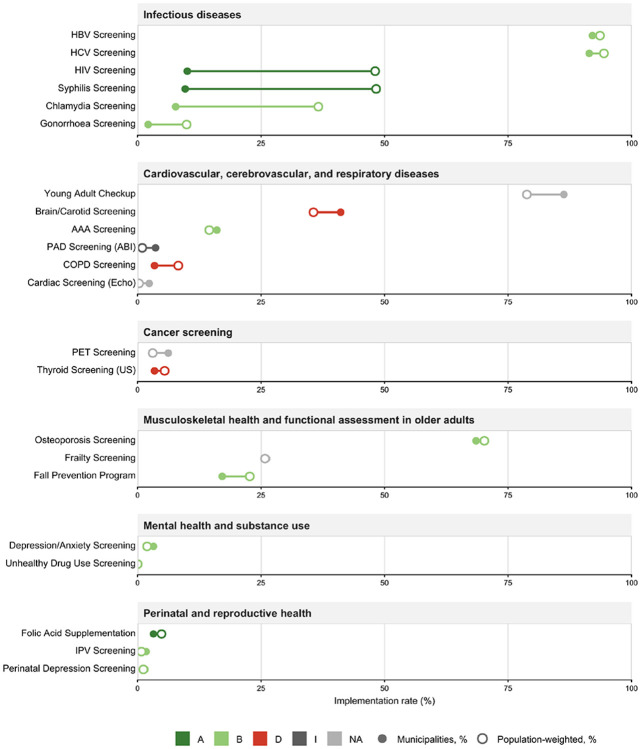
Municipality-count and population-weighted implementation rates of non-mandatory preventive medicine programmes, stratified by disease category and USPSTF recommendation grade. Each programme is shown as a dumbbell: the closed circle indicates the percentage of responding municipalities that reported implementation (n = 467), and the open circle indicates the population-weighted coverage. Programmes are grouped by disease category and labelled with the USPSTF recommendation grade (A, B, D, I, or N/A for ungraded). A wider gap between the two circles indicates greater divergence between municipality-level penetration and population-level coverage; population-weighted coverage exceeding municipality-count rates typically reflects concentration of the service in larger municipalities (as observed for HIV, syphilis, and chlamydia screening), whereas the reverse pattern suggests that the programme is disproportionately implemented in smaller municipalities. USPSTF, US Preventive Services Task Force; HBV, hepatitis B virus; HCV, hepatitis C virus; HIV, human immunodeficiency virus; AAA, abdominal aortic aneurysm; PAD, peripheral artery disease; ABI, ankle–brachial index; US, ultrasonography; COPD, chronic obstructive pulmonary disease; PET, positron emission tomography; IPV, intimate partner violence; N/A, not evaluated by the USPSTF.

Among programmes classified as USPSTF Grade A or B, 10 had municipality-count-based implementation rates below 50%. In contrast, HBV and HCV screening and osteoporosis screening showed relatively high implementation rates. Among programmes classified as USPSTF Grade D, I, or no grade, three had municipality-count-based implementation rates of 10% or above, suggesting gaps between evidence and practice. To highlight these gaps explicitly, programmes with Grade A or B recommendations but implementation rates below 50% are presented in Table 4, and programmes with Grade D, I, or no grade but implementation rates of 10% or above are presented in Table 5. Implementation rates on both a municipality-count and population-weighted basis, stratified by disease category and USPSTF recommendation grade, are visualised as a dumbbell plot (Fig. 1).

Each programme is shown as a dumbbell: the closed circle indicates the percentage of responding municipalities that reported implementation (n = 467), and the open circle indicates the population-weighted coverage. Programmes are grouped by disease category and labelled with the USPSTF recommendation grade (A, B, D, I, or N/A for ungraded). A wider gap between the two circles indicates greater divergence between municipality-level penetration and population-level coverage; population-weighted coverage exceeding municipality-count rates typically reflects concentration of the service in larger municipalities (as observed for HIV, syphilis, and chlamydia screening), whereas the reverse pattern suggests that the programme is disproportionately implemented in smaller municipalities. USPSTF, US Preventive Services Task Force; HBV, hepatitis B virus; HCV, hepatitis C virus; HIV, human immunodeficiency virus; AAA, abdominal aortic aneurysm; PAD, peripheral artery disease; ABI, ankle–brachial index; US, ultrasonography; COPD, chronic obstructive pulmonary disease; PET, positron emission tomography; IPV, intimate partner violence; N/A, not evaluated by the USPSTF.

#### Evidence–Practice Alignment (EPA) score

**Figure 2:**
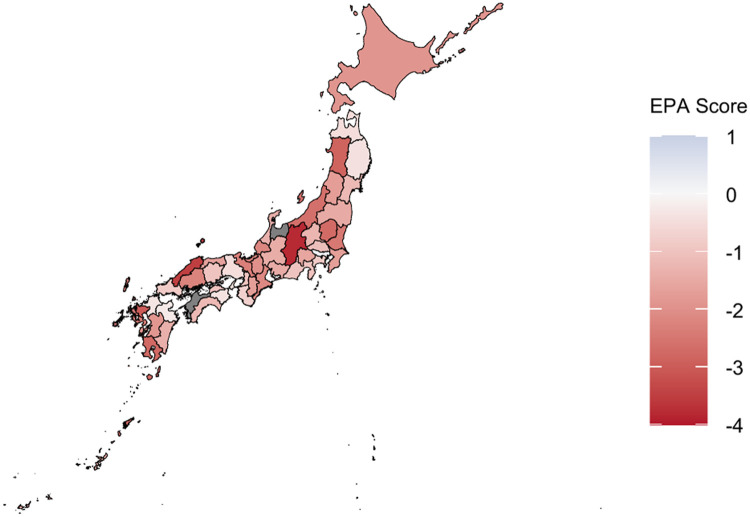
Prefectural Evidence–Practice Alignment (EPA) scores across Japan. Choropleth map displaying the mean EPA score of responding municipalities within each prefecture. The EPA score for each municipality was calculated as a weighted sum of implementation status across all non-mandatory preventive medicine programmes, with weights assigned a priori to reflect the strength of evidence conveyed by each USPSTF grade: A = + 2, B = + 1, D = − 2, and I or ungraded = − 1. Higher scores (blue) indicate greater implementation of recommended services alongside appropriate restraint regarding non-recommended services; lower scores (red) reflect the inverse. Prefectural scores ranged from − 4 to 1. A diverging colour palette centred at zero is used to distinguish prefectures above and below the neutral reference value.

EPA scores were calculated for each municipality and visualised at the prefectural level using a choropleth map (Fig. 2). Scores ranged from − 4 to 1, demonstrating marked heterogeneity in evidence–practice alignment across regions. Prefectures with lower EPA scores, indicating relatively greater implementation of non-recommended or insufficiently evidenced programmes, were widely distributed across the country. In contrast, prefectures with higher scores, reflecting better alignment with evidence-based recommendations, were fewer and appeared to be geographically clustered. Overall, no clear geographic gradient was observed nationwide, suggesting that variation in EPA scores is not solely explained by regional location but may reflect local policy decisions and implementation practices.

Choropleth map displaying the mean EPA score of responding municipalities within each prefecture. The EPA score for each municipality was calculated as a weighted sum of implementation status across all non-mandatory preventive medicine programmes, with weights assigned a priori to reflect the strength of evidence conveyed by each USPSTF grade: A = + 2, B = + 1, D = − 2, and I or ungraded = − 1. Higher scores (blue) indicate greater implementation of recommended services alongside appropriate restraint regarding non-recommended services; lower scores (red) reflect the inverse. Prefectural scores ranged from − 4 to 1. A diverging colour palette centred at zero is used to distinguish prefectures above and below the neutral reference value.

## Discussion

### What this study adds

In this nationwide descriptive cross-sectional survey of Japanese municipalities, we provide the first nationwide municipal-level mapping of implementation patterns for non-mandatory preventive medicine programmes, revealing substantial heterogeneity across domains. Among 1,741 municipalities contacted, 467 returned valid responses (response rate 26.8%). We assessed implementation using two complementary measures: the proportion of municipalities that provided or subsidised each programme and the corresponding population coverage. When we compared municipal practices with USPSTF evidence grades, we found a gap between evidence and implementation. Notably, several programmes graded A or B by the USPSTF were implemented by fewer than half of municipalities—for example, folic acid supplementation (3.2%), syphilis screening (9.6%), chlamydia screening (7.7%), depression/anxiety screening (3.2%), and abdominal aortic aneurysm screening (16.1%). Conversely, a number of programmes graded D or “no grade” were implemented at appreciable levels, including brain/carotid screening (41.1%), young adult health check-ups (86.3%; no grade), and frailty screening (26.1%; no grade).

A further insight was the divergence between municipality-count and population-weighted implementation. Even when relatively few municipalities offered certain STI/HIV services, population-weighted coverage was substantially higher (e.g., HIV screening 10.1% by municipality count vs 48.1% population-weighted), suggesting concentration of such services in larger municipalities.

To visualise municipality-level alignment with evidence-based prevention, we constructed an Evidence–Practice Alignment (EPA) score that summarises implementation patterns across domains.

### Comparison with prior work and interpretation

Internationally, the gap between evidence-based recommendations and real-world adoption is well recognised; simply producing evidence or guidelines rarely ensures consistent uptake without active implementation strategies [18].

In our context, we interpret the under-implementation of multiple A/B-graded services as potentially reflecting not only operational constraints but also limited and uneven dissemination of evidence-based prioritisation into municipal decision-making. Specifically, in Japan it is difficult to argue that a cross-cutting, USPSTF-like centralisation of evidence-graded preventive recommendations is sufficiently institutionalised and disseminated; consequently, municipalities may be less likely to consistently reference graded evidence when selecting preventive medicine programmes. This is a plausible hypothesis rather than a causal conclusion, because our survey did not directly measure municipal decision processes. Nonetheless, it offers a testable explanation for why some highly recommended services remain uncommon while other programmes persist despite weak evidence or potential net harm.

The latter pattern is consistent with concerns about overdiagnosis and low-value screening. Large-scale screening can increase detection of indolent disease without proportional mortality benefit, as illustrated by thyroid cancer overdiagnosis associated with widespread screening in Korea [19] and by evidence that favourable shifts in breast tumour size distributions may be driven largely by additional detection of small tumours with substantial overdiagnosis [20]. At the system level, these dynamics align with the broader challenge of reducing “waste” in healthcare by limiting low-value services rather than cutting beneficial care [21].

### Limitations

This study has several limitations. First, the response rate was modest (26.8%), and non-response bias is possible. Responding municipalities tended to have lower per-capita resident income and fewer general-clinic physicians per 100,000 population; accordingly, results may over-represent municipalities with lower per-capita income and fewer clinic physicians, and under-represent urban or higher-resource municipalities.

Second, “implementation” was defined as municipal provision or subsidisation, and we could not fully capture eligibility criteria, intensity, quality assurance, or participation rates. Therefore, implementation does not necessarily indicate guideline-concordant delivery at the individual level.

Third, USPSTF recommendations are stratified by age, sex, and risk, and are derived from evidence bases reflecting the epidemiological context of the United States. Applying these grades as a benchmark for Japanese municipal programmes involves two important caveats: population-level policy classification does not map directly onto individual-level eligibility criteria, and the prevalence of relevant conditions diverges between the two countries in ways that affect the appropriateness of universal screening—in some cases overstating and in others understating the urgency of implementation. These discordances limit the direct applicability of USPSTF grades, and future studies should incorporate domestically developed evidence-graded frameworks where available. Furthermore, USPSTF grades reflect recommendations current as of March 2025 and may have been updated subsequently; findings should be interpreted with reference to the grades in effect at the time of the survey.

Finally, as a cross-sectional descriptive study, we cannot infer causal determinants of adoption or quantify downstream benefits, harms, or cost consequences.

## Conclusions

In a nationwide municipal survey, we observed substantial variation and clear evidence–practice gaps in Japan’s non-mandatory preventive medicine programmes. Under-implementation of multiple A/B-graded services coexisted with notable implementation of Grade D or ungraded programmes. Population-weighted results further suggested that some evidence-aligned services are concentrated in larger municipalities, raising potential equity concerns.

Our findings support a dual agenda. First, there is a need to strengthen dissemination and implementation support for high-value preventive medicine services, including evaluation of whether limited awareness or uptake of graded evidence contributes to municipal choices in Japan. Second, low-value screening programmes—where harms may outweigh benefits—warrant critical reassessment and, where appropriate, active de-implementation [22]. A structural gap underlying these findings is the absence of a nationally authoritative, evidence-grading body for preventive medicine in Japan analogous to the USPSTF. Establishing such an institution would provide municipalities with a unified and regularly updated reference standard, and represents a necessary policy priority for reducing evidence–practice gaps in Japanese preventive medicine. Future research should examine municipal decision-making to identify modifiable barriers to evidence-aligned prevention.

## Supplementary Material

This is a list of supplementary files associated with this preprint. Click to download.

- Additionalfileslegends.docx
- Additionalfile1.docx
- Additionalfile2.docx
- Additionalfile3.tiff
- Additionalfile4.tiff

## Figures and Tables

**Table 1:** Population size distribution of respondent municipalities (n = 467)

Population size (total population)	n (%)
≥ 1,000,000	5 (1.1)
100,000–999,999	78 (16.7)
10,000–99,999	248 (53.1)
< 10,000	136 (29.1)
**Total**	**467 (100.0)**

Data are presented as number (percentage). Municipalities include cities, towns, villages, and the special wards of Tokyo. Administrative wards of designated cities were excluded as they constitute internal administrative subdivisions rather than independent basic local government units.

**Table 2:** Characteristics of respondent and non-respondent municipalities and standardised mean differences

Characteristic	Responding municipalities (n = 467)	Non-responding municipalities (n = 1,274)	SMD
Aging rate (%)	36.4 (30.1–40.9)	35.5 (29.8–41.7)	0.018
Fiscal capacity index	0.43 (0.28–0.65)	0.44 (0.26–0.66)	0.013
Per-capita resident income (JPY)	3,088,363 (2,894,575–3,354,483)	3,121,105 (2,883,839–3,399,850)	0.124
Hospitals per 100,000 population [n = 358]	8.42 (5.19–12.82)	8.01 (5.23–12.86) [n = 914]	0.001
Physicians per 100,000 population (general clinics) [n = 458]	809.64 (332.73–2,154.56)	1,147.11 (462.28–3,004.44) [n = 1,263]	0.104
Rurality Index for Japan (RIJ, 1–100)	56 (34–79)	54 (30–77)	0.076
Total population	26,694 (7,901–65,217)	21,078 (7,009–59,728)	0.086

Values are presented as median (interquartile range [IQR]). Standardised mean differences (SMDs) were calculated using the mean and standard deviation of each variable, although central tendency and dispersion are displayed as median and IQR to reflect the skewed distributions of municipal-level variables. Bracketed n indicates the number of municipalities with non-missing data for that variable. Baseline characteristics were compared between responding and non-responding municipalities using data obtained from public governmental databases. ∣SMD∣ < 0.10 indicates adequate balance, 0.10–0.20 minor imbalance, and > 0.20 meaningful imbalance.

**Table 3:** Implementation of non-mandatory preventive medicine programmes by Japanese municipalities, by disease category and USPSTF recommendation grade

Preventive programme	USPSTF grade	Municipalities implementing, % (n/N)	Population- weighted (%)	Population covered, n
***Infectious diseases***				
HBV screening	B	92.1 (430/467)	93.6	36,554,094
HCV screening	B	91.4 (427/467)	94.4	36,863,939
HIV screening	A	10.1 (47/467)	48.1	18,778,645
Syphilis screening	A	9.6 (45/467)	48.3	18,858,018
Chlamydia screening	B	7.7 (36/467)	36.6	14,293,149
Gonorrhoea screening	B	2.1 (10/467)	9.9	3,876,682
**Cardiovascular, cerebrovascular, and respiratory diseases**				
Young adult health check-up	No grade	86.3 (403/467)	78.8	30,772,901
Brain/carotid screening	D	41.1 (192/467)	35.6	13,889,906
AAA screening	B	16.1 (75/467)	14.5	5,664,715
PAD screening (ABI)	I	3.6 (17/467)	0.9	369,971
COPD screening	D	3.4 (16/467)	8.2	3,217,836
Cardiac screening (echocardiography)	No grade	2.4 (11/467)	0.3	114,850
**Cancer screening**				
Thyroid screening (ultrasonography)	D	3.4 (16/467)	5.5	2,138,848
Positron emission tomography (PET) screening	No grade	6.2 (29/467)	3.1	1,193,793
**Musculoskeletal health and functional assessment in older adults**				
Osteoporosis screening	B	68.5 (320/467)	70.2	27,420,935
Frailty screening	No grade	26.1 (122/467)	25.8	10,068,792
Fall prevention programme	B	17.1 (80/467)	22.7	8,868,164
**Mental health and substance use**				
***Infectious diseases***				
Depression/anxiety screening	B	3.2 (15/467)	1.9	751,605
Unhealthy drug use screening	B	0.2 (1/467)	0.0	12,782
**Perinatal and reproductive health**				
Folic acid supplementation	A	3.2 (15/467)	4.9	1,895,353
IPV screening	B	1.7 (8/467)	0.8	303,580
Perinatal depression screening	B	1.5 (7/467)	1.2	456,802

This table summarises the implementation rates of non-mandated preventive medicine programmes delivered by Japanese municipalities, stratified by disease category and USPSTF recommendation grade. Implementation was defined as provision or subsidisation of the programmes by a municipality, irrespective of eligibility criteria. Population-weighted rates reflect the proportion of the responding-municipality population living in those implementing municipalities. USPSTF, United States Preventive Services Task Force. Grade A, recommended with high certainty of substantial net benefit; Grade B, recommended with high certainty of moderate net benefit; Grade D, recommended against; Grade I, insufficient evidence; No grade, not evaluated by the USPSTF. HBV, hepatitis B virus; HCV, hepatitis C virus; HIV, human immunodeficiency virus; AAA, abdominal aortic aneurysm; PAD, peripheral artery disease; ABI, ankle–brachial index; COPD, chronic obstructive pulmonary disease; IPV, intimate partner violence. Population covered was estimated using municipal population data.

**Table 4:** Preventive programmes with USPSTF Grade A or B recommendation but municipality-level implementation below 50% This table presents programmes for which the USPSTF assigned a Grade A or B recommendation but fewer than half of responding municipalities reported implementation. Programmes are ordered by ascending implementation rate. This pattern represents a potential evidence-to-practice gap, where evidence-based recommendations have not translated into widespread municipal adoption. USPSTF, United States Preventive Services Task Force. Grade A, recommended with high certainty of substantial net benefit; Grade B, recommended with high certainty of moderate net benefit. IPV, intimate partner violence; AAA, abdominal aortic aneurysm. Implementation was defined as provision or subsidisation of the programme by a municipality, irrespective of eligibility criteria.

Preventive programme	USPSTF grade	Municipalities implementing, % (n/N)
Unhealthy Drug Use Screening	B	0.2 (1/467)
IPV Screening	B	1.7 (8/467)
Gonorrhoea Screening	B	2.1 (10/467)
Depression/Anxiety Screening	B	3.2 (15/467)
Folic Acid Supplementation	A	3.2 (15/467)
Chlamydia Screening	B	7.7 (36/467)
Syphilis Screening	A	9.6 (45/467)
HIV Screening	A	10.1 (47/467)
AAA Screening	B	16.1 (75/467)
Fall Prevention Programme	B	17.1 (80/467)

**Table 5:** Preventive programmes with USPSTF Grade D, I, or No grade but municipality-level implementation of 10% or above

Preventive programme	USPSTF grade	Municipalities implementing, % (n/N)
Young Adult Check-up	No grade	86.3 (403/467)
Brain/Carotid Screening	D	41.1 (192/467)
Frailty Screening	No grade	26.1 (122/467)

This table presents programmes for which the USPSTF assigned a Grade D or no grade, yet 10% or more of responding municipalities reported implementation. Programmes are ordered by descending implementation rate. This pattern represents a potential evidence-to-practice gap, where municipal adoption has outpaced or diverged from current evidence-based guidance. USPSTF, United States Preventive Services Task Force. Grade D, recommended against with moderate or high certainty that the service has no net benefit or that harms outweigh benefits; No grade, not evaluated by the USPSTF. Implementation was defined as provision or subsidisation of the programme by a municipality, irrespective of eligibility criteria.

## Abbreviations

AAA: Abdominal aortic aneurysm
ABI: Ankle–brachial index
COPD: Chronic obstructive pulmonary disease
EPA: Evidence–Practice Alignment
HBV: Hepatitis B virus
HCV: Hepatitis C virus
HIV: Human immunodeficiency virus
IQR: Interquartile range
IPV: Intimate partner violence
MHLW: Ministry of Health, Labour and Welfare
PAD: Peripheral artery disease
PET: Positron emission tomography
RIJ: Rurality Index for Japan
SMD: Standardised mean difference
USPSTF: US Preventive Services Task Force
